# Mechanism-Base Pharmacokinetic–Pharmacodynamic Modeling of Cefquinome Against *Streptococcus suis* Serotype 2 Under Different Inoculum and Susceptibility Conditions

**DOI:** 10.3390/medsci14040505

**Published:** 2026-08-21

**Authors:** Aktham H. Mestareehi

**Affiliations:** 1 Department of Applied Pharmaceutical Sciences and Clinical Pharmacy, Faculty of Pharmacy, Isra University, Amman 11622, Jordan; aktham.mestareehi@med.wayne.edu; 2 Department of Pharmaceutical Sciences, Eugene Applebaum College of Pharmacy and Health Sciences, Wayne State University, Detroit, MI 48201, USA

**Keywords:** cefquinome, *Streptococcus suis* serotype 2, ex vivo experiment, model- and simulation-based approach, pharmacokinetics (PK), pharmacodynamics (PD)

## Abstract

**Background**: *Streptococcus suis* serotype 2 is a major zoonotic pathogen responsible for severe systemic infections in pigs and humans, including septicemia, meningitis, and high mortality outcomes. Cefquinome, a fourth-generation β-lactam antibiotic widely used in veterinary medicine, is commonly applied for the treatment of *S. suis* infections. However, optimized dosing strategies remain insufficiently defined, particularly under conditions of varying bacterial burden, inoculum size, and reduced susceptibility or resistance phenotypes. These factors may significantly alter pharmacodynamic responses and compromise the predictive value of conventional MIC-based approaches. **Objectives**: This study aimed to characterize the pharmacokinetics (PK) and pharmacodynamics (PD) of cefquinome against *S. suis* serotype 2 using an integrated ex vivo serum time-kill experiments and semi-mechanistic PK/PD modeling. A secondary objective was to evaluate optimized dosing regimens across different inoculum levels and susceptibility phenotypes, including a cefquinome-resistant mutant. **Methods**: Cefquinome pharmacokinetics following intramuscular administration at 2 and 4 mg/kg in piglets were described using a two-compartment model. Dose proportionality, exposure linearity, and clearance parameters were assessed. Ex vivo serum time-kill experiments were conducted using a parental strain and a cefquinome-resistant mutant (M1) under normal-inoculum (NI), high-inoculum (HI), and mutant/resistant (MS) conditions. A semi-mechanistic PK/PD model incorporating logistic bacterial growth, sigmoidal Emax killing, nutrient limitation, and a time-delay function was developed to describe dynamic bacterial responses. Model parameters (k_0_, k_max_, EC_50_) were estimated using nonlinear least-squares regression (Scientist v2.0), and simulations were performed by integrating time-varying PK input functions. **Results**: Cefquinome demonstrated linear pharmacokinetics with dose-proportional increases in Cmax and AUC between 2 and 4 mg/kg, with comparable clearance across doses. Ex vivo studies revealed time-dependent antibacterial activity with a pronounced inoculum effect. Higher bacterial burdens significantly reduced bactericidal efficiency and promoted regrowth during declining drug exposure. No tested concentrations achieved ≥3-log_10_ killing in HI or MS conditions, whereas the NI group achieved a maximal reduction of 3.5-log_10_ CFU/mL. MIC values in serum and medium were consistent (0.03, 0.06, and 0.24 µg/mL for NI, HI, and MS, respectively), indicating minimal protein binding influence. The semi-mechanistic model accurately described observed bacterial dynamics (R^2^ > 0.99; MSC > 1.5), capturing delayed drug effects, inoculum-dependent growth suppression, and regrowth phenomena. Growth rates were reduced under serum conditions, reflecting nutrient limitation. Importantly, inoculum size exerted a stronger impact on pharmacodynamic outcomes than resistance phenotype, as reflected by reductions in kmax and increases in EC_50_ under HI conditions. Although %T>MIC exceeded conventional β-lactam targets (>40%) in most regimens, MIC-based indices poorly correlated with observed dynamic killing responses. **Conclusions**: Cefquinome exhibited time-dependent antibacterial activity against *S. suis* serotype 2, strongly modulated by inoculum size and reduced susceptibility. The developed semi-mechanistic PK/PD model provided robust prediction of bacterial time-kill behavior and outperformed MIC-based metrics in guiding dose optimization. Simulation results support 2 mg/kg every 24 h for normal infections and 2 mg/kg every 12 h for high-inoculum or less susceptible infections, emphasizing the value of model-informed dosing strategies for optimizing β-lactam therapy.

## 1. Introduction

*Streptococcus suis* (*S. suis*) is an important Gram-positive bacterium and an emerging zoonotic pathogen of major global importance, particularly in swine production systems, where it contributes significantly to economic losses and animal welfare concerns [1]. In humans, it is recognized as one of the most important causes of bacterial meningitis in individuals with occupational exposure to pigs or pork-derived products. Clinically, it manifests predominantly as sepsis, meningitis, and septic shock, with serotype 2 consistently identified as the most virulent and epidemiologically dominant strain responsible for severe invasive disease [2,3]. The high morbidity and mortality associated with these infections, together with their increasing global distribution, highlight the urgent need for optimized and evidence-based antimicrobial treatment strategies.

β-lactam antibiotics remain the first-line therapeutic option for *S. suis* infections due to their well-established efficacy and favorable safety profile. However, the emergence and gradual increase in reduced susceptibility and resistance to penicillin and fourth-generation cephalosporins have raised significant concerns regarding the long-term reliability of standard treatment regimens [4]. This evolving resistance landscape underscores the importance of reassessing current therapeutic strategies and preserving the effectiveness of available agents through rational and optimized dosing. Cefquinome, a fourth-generation cephalosporin exclusively used in veterinary medicine, exhibits broad-spectrum activity against both Gram-positive and Gram-negative pathogens, including *S. suis* [5,6]. Its enhanced stability against β-lactamases and favorable pharmacodynamic profile make it a valuable candidate for treating resistant bacterial infections in livestock. However, despite its clinical utility, limited data is available regarding its efficacy against *Streptococcus suis*. With elevated β-lactam resistance, particularly under conditions that mimic a clinically relevant infection burden. Consequently, preserving the antimicrobial activity of cefquinome requires the development of optimized dosing regimens that not only ensure therapeutic efficacy but also minimize the selection and enrichment of resistant subpopulations [7].

The relationship between antimicrobial exposure and bacterial killing is commonly described using minimal inhibitory concentration (MIC)-based pharmacokinetic/pharmacodynamic (PK/PD) indices, including %ƒT>MIC, ƒC_max_/MIC, and ƒAUC24h/MIC [8,9]. β-lactams are classically categorized as time-dependent antibiotics, with efficacy primarily driven by the duration that free drug concentrations remain above the MIC (%ƒT>MIC), whereas concentration-dependent agents such as aminoglycosides and fluoroquinolones exhibit activity more closely associated with peak concentration (ƒC_max_/MIC) or total exposure (ƒAUC24h/MIC), respectively [10]. Although these indices have been instrumental in defining dosing strategies, they inherently simplify complex bacterial dynamics by relying on static MIC endpoints.

In a previous study, researchers demonstrated that the magnitude of %ƒT>MIC required to achieve bactericidal activity of cefquinome against *S. suis* serotype 2 was 37 ± 4.58% and 52.03 ± 6.71% for standard (10^6^ CFU/thigh) and high (10^8^ CFU/thigh) inoculum sizes, respectively, indicating an inoculum-dependent effect on drug efficacy [11]. However, MIC-based PK/PD approaches are limited by their endpoint nature, as they do not account for dynamic bacterial regrowth, adaptive responses, or temporal fluctuations in antimicrobial exposure during therapy. To overcome these limitations, model-and-simulation-based PK/PD approaches integrate time-kill kinetics with pharmacokinetic data to directly characterize drug–bacteria interactions over time. This framework enables a more mechanistic understanding of antibacterial activity and provides a stronger basis for dose selection and regimen optimization [12,13]. When combined with in vivo pharmacokinetic parameters, such models allow for more accurate translation of experimental findings into clinically relevant dosing strategies.

Ex vivo infection models further enhance translational relevance by incorporating host-related factors such as serum protein binding, immune components, and physiological drug distribution, thereby providing a more clinically representative assessment of antimicrobial activity, particularly in systemic infections such as sepsis [14].

The aim of the present study was therefore to apply a model-and-simulation-based PK/PD framework, integrated with an ex vivo infection model, to systematically evaluate and optimize cefquinome dosing regimens against *S. suis* serotype 2 under different infection conditions. Specifically, we aimed to develop a robust mathematical model by fitting ex vivo time-kill data; compare the bactericidal activity of cefquinome across different inoculum sizes and resistant phenotypes; and simulate and predict the pharmacodynamic outcomes of multiple dosing regimens to support rational optimization of antimicrobial therapy.

## 2. Materials & Methods

### 2.1. Bacterial Strains and Determination of MIC

*Streptococcus suis* serotype 2 ATCC 43765 (VA 20110, United States, 1 lyophilized vial per unit order) was purchased from the American Type Culture Collection (ATCC) (Manassas, VA, USA). A cefquinome-resistant mutant strain (M1) derived from the parental strain ATCC 43765 strain was obtained from a previously established laboratory strain collection and was included in the present study as a reduced-susceptibility phenotype [15]. M1 was not generated or genetically sequenced in the present study. Minimum inhibitory concentrations (MICs) of cefquinome against both strains were determined using agar and broth dilution methods to assess antimicrobial susceptibility and potential serum protein binding effects, in accordance with Clinical and Laboratory Standards Institute (CLSI) guidelines [16,17]. Briefly, ATCC 43765 was cultured overnight in tryptic soy broth (TSB) at 37 °C with vigorous shaking. A 100 µL aliquot of the culture (~10^9^ CFU/mL) was plated onto tryptic soy agar (TSA) containing cefquinome at 2×, 4×, 8×, and 16× MIC and incubated at 37 °C for 48–72 h. Colonies surviving on 2× and 4× MIC plates were isolated and re-cultured on TSA containing the corresponding antibiotic concentrations to confirm phenotype stability. A stable mutant strain (MIC = 0.24 µg/mL) was obtained and stored at −80 °C in 20% glycerol until use. The parental strain exhibited an MIC of 0.03 µg/mL, whereas M1 exhibited an MIC of 0.24 µg/mL, corresponding to an approximately eight-fold increase in cefquinome MIC. The stability of the resistant phenotype was assessed following ten serial passages on antibiotic-free medium before subsequent experiments. Because whole-genome or targeted molecular characterization was not performed in the present study, specific mutations or resistance genes responsible for the M1 phenotype cannot be assigned.

### 2.2. Chemicals and Reagents

Cefquinome sulfate (purity 99.5%) was purchased from Sigma (St. Louis, MO, USA). Tryptic soy broth (TSB) and tryptic soy agar (TSA) were obtained from Sigma-Aldrich (USA). Defibrinated sheep blood (5%) was supplied by Fisher Scientific (Marietta, OH, USA). All reagents used for analysis in this experiment were HPLC or analytical grade and obtained from commercial sources, including acetonitrile (ACN) and methanol (MeOH) (Fisher Scientific, Marietta, OH, USA), HPLC-grade water (Fisher Scientific, Marietta, OH, USA), and trifluoroacetic acid (TFA; Sigma; USA).

### 2.3. Animals and Ethical Statement

Eight healthy crossbred piglets (four males and four females; body weight 19.62–22.82 kg) were enrolled in the pharmacokinetic study. All eight animals contributed to the crossover pharmacokinetic evaluation. The animals were housed under standard controlled environmental conditions and fed an antibiotic-free diet twice daily, with ad libitum access to water. The animals were maintained in compliance with the guidelines of the American Association for Accreditation of Laboratory Animal Care [18]. All animals remained clinically healthy throughout the experimental period. The study protocol was approved by the Laboratory Animal Welfare and Ethics Committee of the Isra University (Approval No.: SREC/26/06/217) and conducted in accordance with National Research Council guidelines for the care and use of laboratory animals.

### 2.4. Cefquinome Dose Used

Cefquinome was administered intramuscularly at 2 and 4 mg/kg. The 2 mg/kg dose was selected because it represents a commonly used and clinically established cefquinome dose in swine and has been extensively evaluated in previous porcine PK and PK/PD studies. Previous investigations of cefquinome against *S. suis* have also used 2 mg/kg by the intramuscular route for PK/PD evaluation. The 4 mg/kg dose was selected as a two-fold higher dose to characterize dose proportionality and to evaluate whether increased systemic exposure would translate into improved pharmacodynamic activity under normal, high-inoculum, and reduced-susceptibility conditions. Thus, the two doses were selected to provide a clinically relevant reference exposure and a higher exposure scenario for PK/PD comparison.

### 2.5. Pharmacokinetic Study Design

A randomized, two-period, two-sequence crossover design was used to characterize the pharmacokinetics of cefquinome following intramuscular (IM) administration. Eight healthy crossbred piglets (four males and four females; body weight, 19.62–22.82 kg) were randomly assigned to two treatment sequences (*n* = 4 per sequence). Each animal received both cefquinome doses, 2 and 4 mg/kg, during separate experimental periods. Animals assigned to Sequence 1 received cefquinome at 2 mg/kg during Period 1 and 4 mg/kg during Period 2, whereas animals assigned to Sequence 2 received 4 mg/kg during Period 1 and 2 mg/kg during Period 2.

A 10-day washout period was implemented between treatment periods to ensure adequate elimination of cefquinome and minimize potential carryover effects before administration of the alternate dose. Blood samples were collected from the cranial vena cava at 0 (Predose), 0.083 (5 min), 0.25, 0.5, 1, 2, 4, 6, 8, 10, 12, and 24 h after each cefquinome administration. Blood samples were allowed to clot and were subsequently centrifuged to obtain serum. The separated serum was stored at −80 °C until analysis.

Each animal therefore contributed pharmacokinetic data to both treatment periods, resulting in eight animals contributing data to each dose group. Serum cefquinome concentrations were quantified using the validated LC-ESI-MS/MS method described below. The resulting concentration–time profiles were used to estimate pharmacokinetic parameters and to generate the pharmacokinetic input functions for subsequent PK/PD modeling and simulation.

### 2.6. Quantification of Cefquinome and Pharmacokinetic Analysis

Serum concentrations of cefquinome were quantified using liquid chromatography–electrospray ionization tandem mass spectrometry (LC-ESI-MS/MS). Analyses were performed using an Agilent 1200 HPLC system (Agilent Technologies, Palo Alto, CA, USA) coupled to an AB Sciex API 4000 triple quadrupole mass spectrometer (AB Sciex, Foster City, CA, USA) equipped with an electrospray ionization source operating in positive ion mode. Chromatographic separation was achieved on a Waters Symmetry C_18_ column (2.1 × 50 mm, 3.5 μm). The injection volume was 5 μL, and the column temperature was maintained at 35 °C. The mobile phase consisted of acetonitrile (A) and 0.1% formic acid in water containing 2 mM ammonium acetate (B). Gradient elution was performed at a flow rate of 200 μL/min as follows: 0.0–1.5 min, 5–60% A; 1.5–4.5 min, 60% A; 4.5–5.5 min, 60–5% A; and 5.5–11.0 min, 5% A. The total run time was 11 min.

Mass spectrometric parameters were set as follows: ionspray voltage, 4000 V; curtain gas, 20 psi; nebulizer gas, 55 psi; collision gas, 20 psi; and source temperature, 500 °C. Multiple reaction monitoring (MRM) transitions of *m*/*z* 529.3→134.1 and 529.3→396.2 were monitored in positive ion mode. The lower limit of quantification (LLOQ) was 0.001 μg/mL. Calibration curves were linear over the concentration range of 0.001–0.5 μg/mL (R^2^ > 0.999). The intra- and inter-day precision values were <12.75% and <17.85%, respectively, whereas the mean extraction recovery exceeded 88%. Pharmacokinetic parameters were calculated using WinNonlin version 5.2 (Pharsight Corporation, Mountain View, CA, USA). Model selection was based on the Akaike information criterion (AIC), and a two-compartment model was identified as the best fit for the concentration–time data.

### 2.7. Ex Vivo Serum Time-Kill Assay

The ex vivo antibacterial activity of cefquinome was evaluated using serum samples collected from the piglets following cefquinome administration. Serum collected at 0, 0.25, 0.5, 1, 2, 4, 6, 8, 10, and 12 h post-dose was used to reproduce the time-varying cefquinome exposure observed in vivo. Serum collected immediately before drug administration (0 h) served as drug-free control.

A stationary-phase culture of *S. suis* serotype 2 was diluted and inoculated into serum to obtain final bacterial densities of approximately 5 × 10^5^ CFU/mL for the normal-inoculum (NI) condition and 5 × 10^7^ CFU/mL for the high-inoculum (HI) condition. The cefquinome-resistant mutant M1 was evaluated at an initial density of approximately 1 × 10^5^ CFU/mL and designated the mutant-strain (MS) condition. The inoculated serum samples were incubated at 37 °C under the appropriate culture conditions, and viable bacterial counts were determined at 0, 2, 4, 6, 8, 10, and 12 h.

For each experimental condition, serum obtained from individual piglets was treated as an independent biological replicate. Eight independent serum samples were evaluated per treatment conditions. At each sampling time, 10 µL aliquots were serially diluted in sterile saline and plated onto TSA. Plates were incubated at 37 °C for 24 h, after which colonies were counted and expressed as CFU/mL. Drug-free serum containing the corresponding bacterial inoculum served as the growth control. Appropriate plating controls were included to verify bacterial recovery and exclude contamination. The lower limit of detection was approximately 10 CFU/mL.

Cefquinome concentrations measured in the serum samples represented total serum concentrations and were used as the exposure input for the PK/PD simulations. The unbound fraction of cefquinome was not directly measured in the experimental serum matrix; therefore, free-drug concentrations were not independently quantified.

### 2.8. Model- and Simulation-Based PK/PD Model Building

Mathematical modeling of serum time-kill data was performed using nonlinear least-squares regression implemented in Scientist v.2 (Micromath, Salt Lake City, UT, USA) [19]. Model selection was guided by the model selection criterion (MSC) provided by the software, and the model with the highest MSC value was selected as the best fit.

A semi-mechanistic PK/PD model integrating bacterial growth dynamics and drug-induced killing was developed. The model couples a logistic growth function with a sigmoidal Emax killing function and is described as follows:

$$\frac{dN}{dt}=k_{0}\left(1-\frac{N}{N_{max}}\right)N-\frac{k_{max}C^{h}}{EC_{50}^{h}+C^{h}}\left(1-e^{-rt}\right)N$$

where $\frac{dN}{dt}$ represents the change in bacterial density over time. The first term describes bacterial growth in the absence of antibiotic exposure following a logistic growth model. Here, $k_{0}$ (h^−1^) is the intrinsic bacterial growth rate constant, N (CFU/mL) is the bacterial population at time t, and $N_{max}$ represents the carrying capacity determined by nutrient limitation and environmental constraints.

The second term describes antibiotic-induced bacterial killing using a sigmoidal E_max_ relationship. In this function, $k_{max}$ (h^−1^) represents the maximal bacterial killing rate, $EC_{50}$ (µg/mL) is the cefquinome concentration producing 50% of the maximal effect, C (µg/mL) is the free drug concentration at time t, and h is the Hill coefficient describing the steepness of the concentration–response relationship. The term $\left(1-e^{-rt}\right)$ is an empirical time-delay function introduced to account for the delayed onset of cefquinome-mediated bactericidal activity, where r is the delay rate constant.

Once the model was parameterized and validated against the ex vivo time-kill data, it was used to simulate bacterial kill dynamics under different cefquinome exposure scenarios. This was achieved by substituting the time-dependent drug concentration term with in vivo pharmacokinetic profiles derived from the two-compartment model, thereby enabling prediction of antibacterial effects under clinically relevant dosing regimens.

The pharmacokinetic model describing cefquinome disposition following once- or twice-daily intramuscular administration is given by:

$$\frac{dC}{dt}=\frac{Ae^{-\alpha t}\left(1-e^{-n\alpha \tau }\right)}{\left(1-e^{-\alpha \tau }\right)}+\frac{Be^{-\beta t}\left(1-e^{-n\beta \tau }\right)}{\left(1-e^{-\beta \tau }\right)}-\frac{(A+B)e^{-k_{a}t}\left(1-e^{-nk_{a}\tau }\right)}{\left(1-e^{-k_{a}\tau }\right)}$$

where t denotes time; A and B are intercept terms corresponding to the distribution and elimination phases, respectively; α and β are hybrid rate constants describing distribution and elimination phases; and $k_{a}$ is the first-order absorption rate constant. The parameter n represents the number of administered doses, and τ is the dosing interval. This superposition approach allows simulation of multiple dosing regimens under steady-state and non-steady-state conditions.

## 3. Results

### 3.1. Bacterial Susceptibility and Mutant Selection

The parental *Streptococcus suis* serotype 2 ATCC 43765 strain and its cefquinome-resistant derivative M1 were successfully characterized by antimicrobial susceptibility testing. The cefquinome MIC increased from 0.03 µg/mL in the parental strain to 0.24 µg/mL in M1, representing an approximately eight-fold reduction in susceptibility. The elevated MIC phenotype remained stable following ten serial passages in antibiotic-free medium, supporting the phenotypic stability of M1 for subsequent ex vivo PK/PD experiments and modeling. Because whole-genome sequencing or targeted genetic analysis was not performed in the present study, the specific genetic alteration(s) responsible for the increased cefquinome MIC in M1 could not be determined. Therefore, M1 is referred to throughout this study as a cefquinome-resistant or reduced-susceptibility mutant based on its observed antimicrobial susceptibility phenotype, without assigning a specific underlying resistance mechanism.

### 3.2. Pharmacokinetics

**Figure 1:**
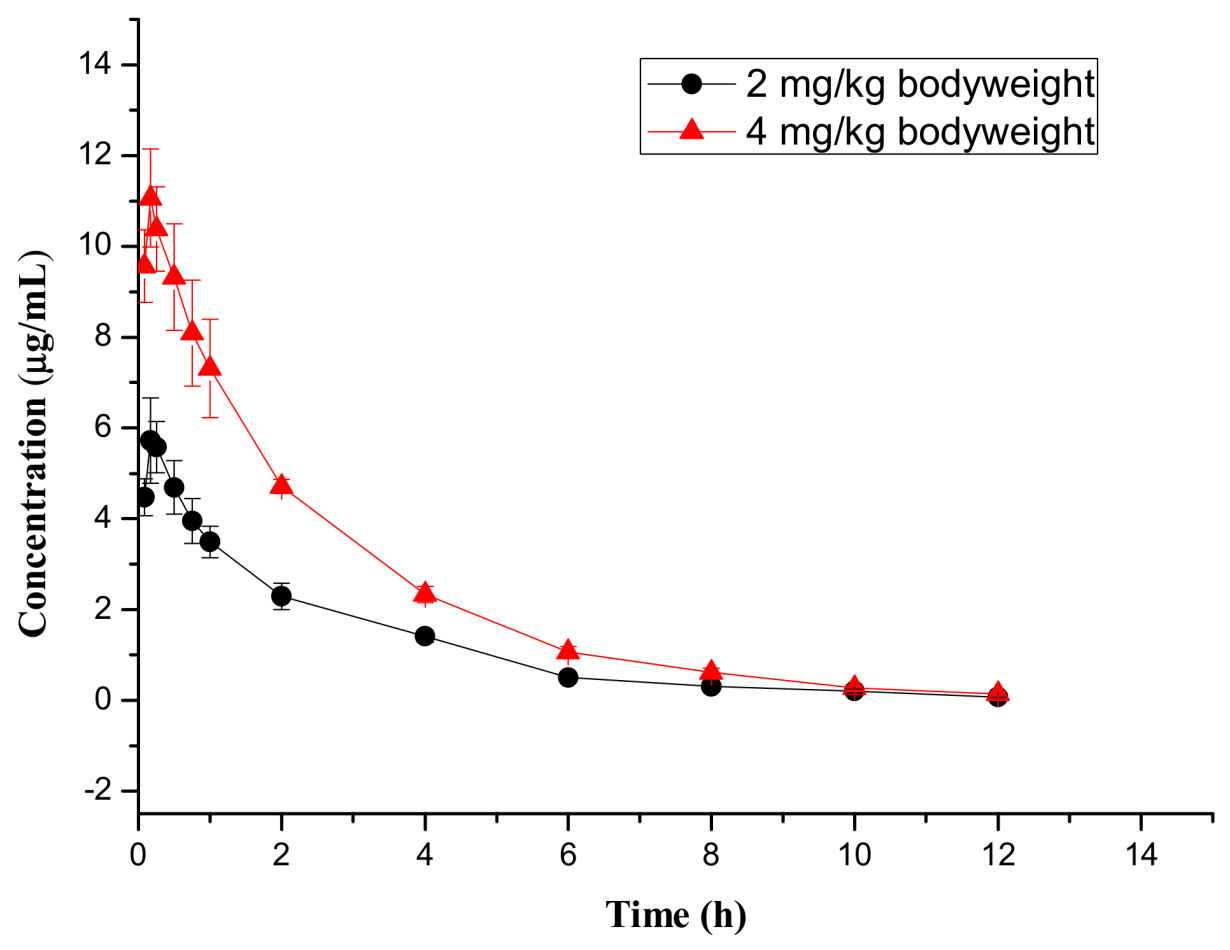
Arithmetic plots of cefquinome serum concentration–time profiles following intramuscular (IM) administration at doses of 2 and 4 mg/kg (*n* = 8). Data are presented as mean ± standard deviation (SD).

The pharmacokinetic profiles of cefquinome following intramuscular administration were best described by a two-compartment model for both 2 and 4 mg/kg dosing regimens. Model selection based on Akaike information criterion confirmed this structure as the optimal fit. Drug disposition was characterized by rapid absorption and biexponential decline, consistent with distinct distribution and elimination phases. Pharmacokinetic parameters were comparable between doses, with nearly identical clearance values observed. As expected under linear kinetics, systemic exposure increased proportionally with dose, with the 4 mg/kg group exhibiting approximately two-fold higher Cmax and AUC compared with the 2 mg/kg group (Figure 1; Table 1). These parameters were subsequently used as input functions for PK/PD simulation.

### 3.3. Ex Vivo Susceptibility and Serum Time-Kill Dynamics

**Figure 2:**
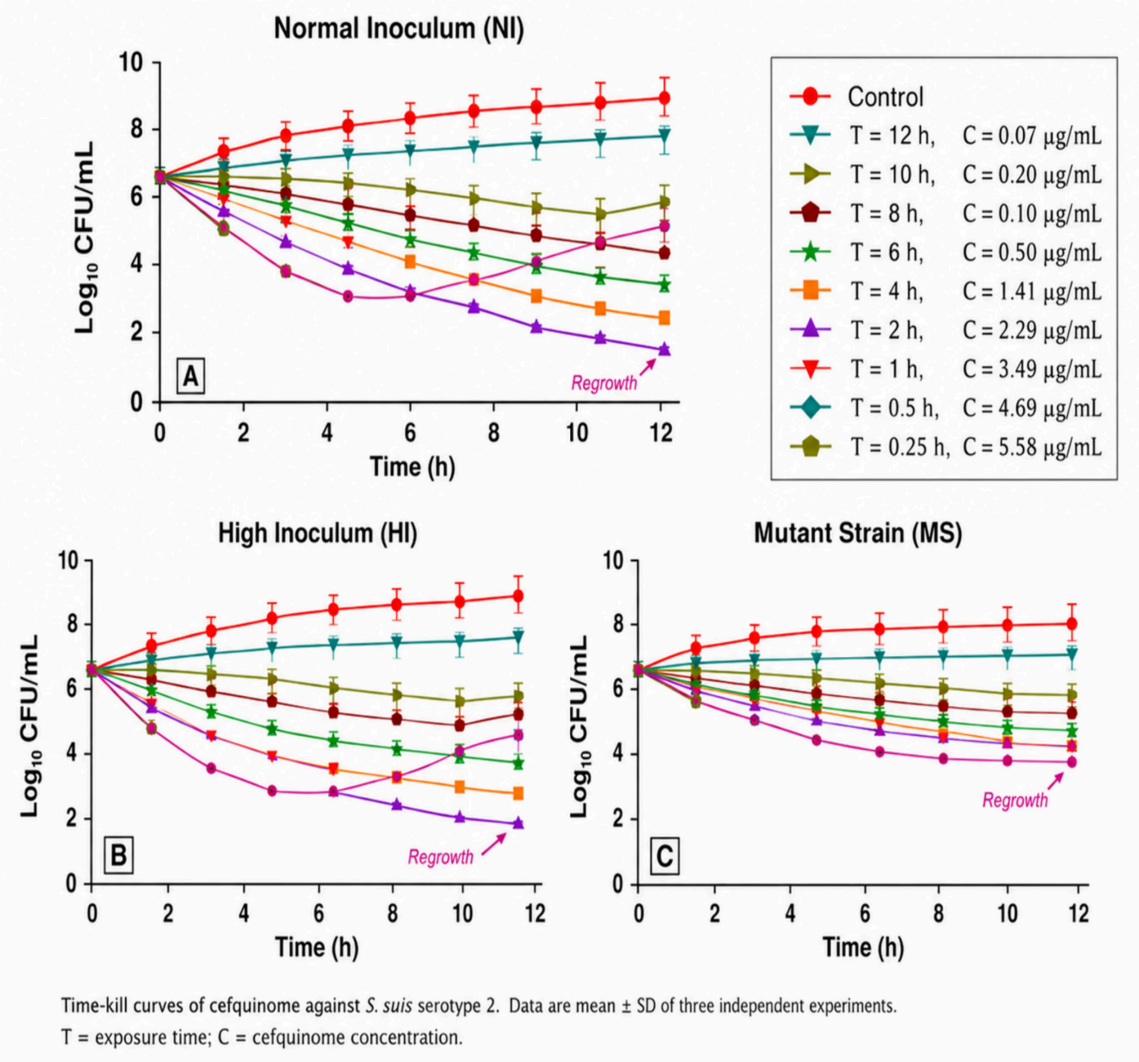
Time-kill curves of different cefquinome concentrations (corresponding to mean serum concentrations at different time points) against *Streptococcus suis* serotype 2 under normal-inoculum (MIC = 0.03 µg/mL) (**A**), high-inoculum (MIC = 0.06 µg/mL) (**B**), and mutant strain (MIC = 0.24 µg/mL) (**C**) conditions. Data are presented as mean ± standard deviation (SD). Dots represent the observed data, whereas lines represent the simulated data.

MIC values of cefquinome against *S. suis* serotype 2 were identical in culture medium and serum for all bacterial populations, with values of 0.03, 0.06, and 0.24 µg/mL for the NI, HI, and MS groups, respectively. The comparable MIC values obtained in the two matrices suggest that the serum matrix did not substantially alter the apparent antibacterial susceptibility of the tested strains under the conditions evaluated. Ex vivo time-kill experiments (Figure 2; Table 2) showed that, in the absence of cefquinome exposure, bacteria underwent a short adaptation phase followed by rapid growth, reaching an apparent stationary phase at approximately 8 h. Cefquinome demonstrated time-dependent antibacterial activity, with the magnitude and duration of bacterial killing influenced by both drug exposure and bacterial inoculum. At the lowest cefquinome concentration tested (0.07 µg/mL), regrowth occurred in the NI group, whereas predominantly bacteriostatic activity was observed in the HI group. In the NI group, the maximum observed antibacterial effect resulted in approximately a 3.5-log10 reduction in bacterial counts within 4 h. In contrast, none of the tested cefquinome concentrations produced ≥3-log10 bacterial killing in either the HI or MS groups.

Overall, increasing cefquinome concentrations beyond the lower exposure levels produced relatively limited additional bacterial killing, indicating a plateau in the concentration–effect relationship within the range evaluated. This finding is consistent with the predominantly time-dependent pharmacodynamic characteristics of β-lactam antibiotics, for which antibacterial efficacy is generally more closely associated with the duration of drug exposure above the organism-specific susceptibility threshold than with increasing peak concentrations. A clear inoculum effect was also observed in the parental strain, with higher initial bacterial burdens associated with reduced killing and more pronounced bacterial regrowth.

**Figure 3:**
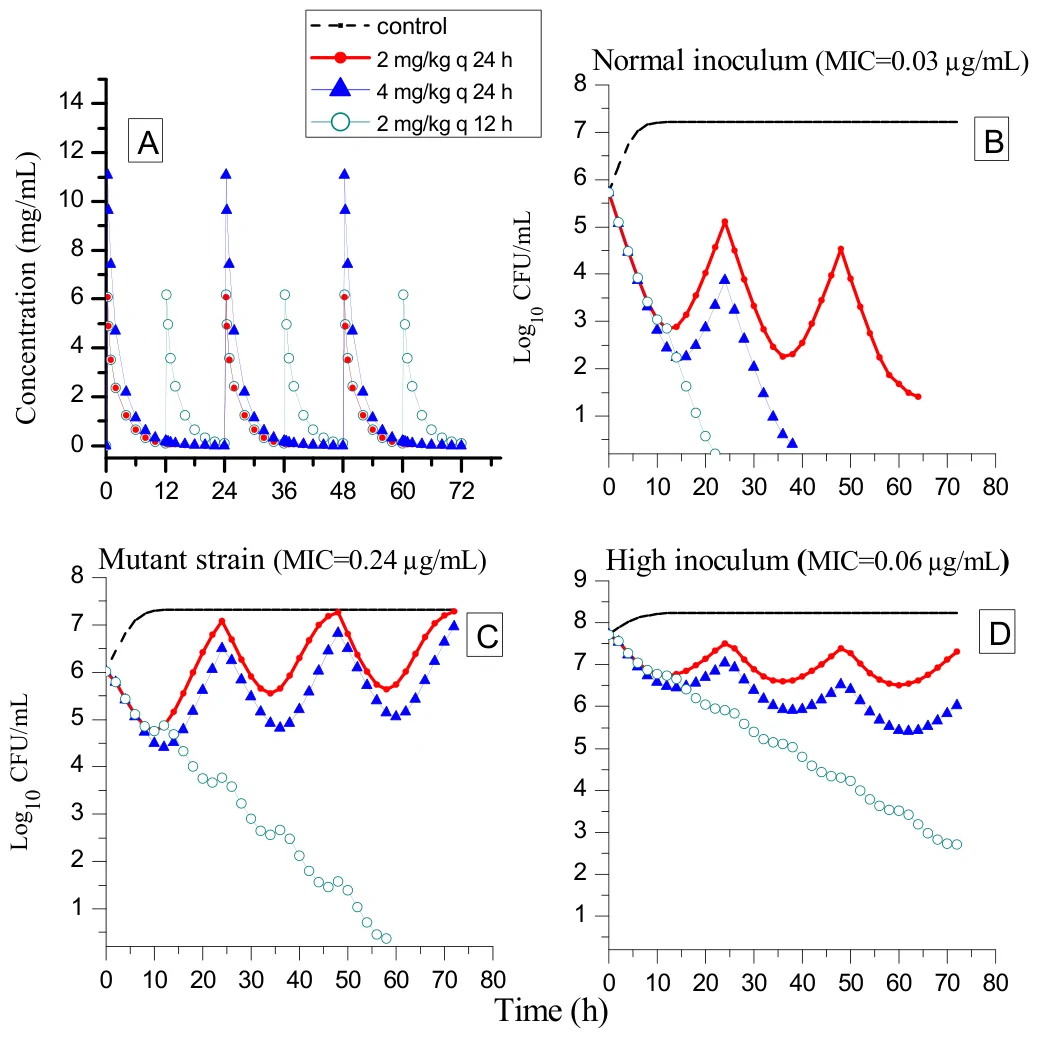
Predicted serum cefquinome concentration–time profiles (**A**) and expected antibacterial outcomes against normal inoculum (**B**), mutant strain (**C**), and high inoculum (**D**). Control growth in the absence of drug is represented by black circles. Simulated plasma concentration–time profiles for multiple cefquinome dosing regimens are shown as follows: 2 mg/kg every 24 h (q24h; red circles), 4 mg/kg q24h (blue solid triangles), and 2 mg/kg every 12 h (q12h; green open circles).

At the lowest cefquinome concentration tested (0.07 µg/mL), an initial reduction in bacterial counts followed by early regrowth was observed in the NI group (Figure 2, arrow). Under the same exposure condition, the HI group showed predominantly bacteriostatic activity. These short-term experimental observations primarily characterize the initial drug–bacteria interaction and were used to inform estimation of the PK/PD parameters. More pronounced and sustained regrowth associated with declining cefquinome exposure was observed in the longer-term PK/PD simulations presented in Figure 3.

### 3.4. Simulation of Dosing Regimens and Bacterial Kill Dynamics

Model-based simulations demonstrated that cefquinome efficacy was highly dependent on both exposure and inoculum size. At lower inoculum conditions, standard dosing regimens achieved sustained bacterial suppression, whereas high-inoculum conditions required increased exposure to achieve comparable bacterial reduction. Simulations further indicated that suboptimal exposure resulted in rapid bacterial regrowth once drug concentrations fell below the pharmacodynamic threshold, consistent with the time-dependent killing behavior incorporated in the model structure.

In the resistant mutant strain, simulated killing curves showed limited response even under higher exposure scenarios, confirming the impact of reduced susceptibility on pharmacodynamic outcomes.

### 3.5. Ex Vivo Serum Antibacterial Activity and Inoculum Effect

The ex vivo time-kill data were adequately described using a semi-mechanistic model incorporating logistic bacterial growth, a sigmoidal E_max_ killing function, nutrient limitation, and a time-delay component. Model predictions closely matched the observed bacterial counts, with R^2^ > 0.99 and MSC > 1.5 (Figure 2). The estimated net bacterial growth rates were 0.67 ± 0.09 h^−1^, 0.32 ± 0.03 h^−1^, and 0.55 ± 0.03 h^−1^ for the NI, HI, and MS groups, respectively. A lag-phase parameter (γ) was identified for the MS and HI groups. The NI group showed greater cefquinome susceptibility, characterized by a higher maximum killing rate (k_max_ = 1.39 ± 0.24 h^−1^) and lower EC_50_ (0.08 ± 0.06 µg/mL) than the other groups.

### 3.6. PK/PD Modeling of Ex Vivo Data

Time-kill data were successfully described using a semi-mechanistic model incorporating logistic bacterial growth, a sigmoidal E_max_ killing function, nutrient limitation, and a time-delay component. Model simulations closely matched observed bacterial counts, with excellent goodness-of-fit (R^2^ > 0.99; MSC > 1.5) as illustrated in Figure 2. The net bacterial growth rates were similar for the NI and MS groups (0.67 ± 0.09 h^−1^ and 0.55 ± 0.03 h^−1^, respectively), whereas the HI group exhibited reduced growth (0.32 ± 0.03 h^−1^). A lag-phase parameter (γ) was identified only in the MS and HI groups, reflecting a delayed antimicrobial effect. The NI group demonstrated greater susceptibility, with a higher maximal killing rate (k_max_ = 1.39 ± 0.24 h^−1^) and lower EC_50_ (0.08 ± 0.06 µg/mL) compared with the other groups.

### 3.7. Integration of Pharmacokinetics with Pharmacodynamics

In vivo pharmacokinetic profiles were integrated directly into the PK/PD model to enable dynamic simulation of bacterial responses under clinically relevant dosing regimens. Serum concentration–time data generated by the two-compartment model were incorporated into the killing function C(t), allowing prediction of bacterial burden over time across different exposure scenarios. This mechanistic integration successfully linked systemic drug exposure to antibacterial effect, enabling simulation of cefquinome activity under varying doses, dosing intervals, and infection intensities.

### 3.8. PK/PD Simulation Analysis

The percentage of time that free drug concentrations exceeded the MIC (%T>MIC) exceeded 50% for most regimens, except in the MS group receiving 2 mg/kg every 24 h (37.5%) and 4 mg/kg every 24 h (45.8%) as shown in Table 3. However, MIC-based predictions did not fully align with model-based dynamic outcomes. Simulated concentration–time profiles (Figure 3A) demonstrated that more frequent dosing improved systemic exposure. Corresponding bacterial kill curves (Figure 3B–D) showed that with 2 mg/kg every 24 h, only limited early killing occurred, followed by regrowth in all groups. The NI group eventually achieved a 4-log10 reduction at 72 h, whereas the MS group exhibited treatment failure and the HI group showed only bacteriostatic effects. Increasing the dose to 4 mg/kg every 24 h improved outcomes in the NI group, achieving eradication by 48 h, with partial improvement in the HI group and no meaningful change in the MS group. In contrast, shortening the dosing interval to every 12 h markedly enhanced antibacterial efficacy across all infection models, achieving bactericidal effects at 24 h (NI), 48 h (MS), and 60 h (HI), respectively. Overall, 2 mg/kg every 24 h was sufficient for low-inoculum infections, whereas high-inoculum or resistant infections required more frequent dosing (2 mg/kg every 12 h) to achieve optimal antibacterial activity.

### 3.9. Model Performance and Predictive Capability

The integrated PK/PD model reliably reproduced observed ex vivo time-kill data and accurately predicted bacterial dynamics under multiple exposure conditions. The model captured key system behaviors, including: (1) bacterial growth and population expansion in the absence of effective antimicrobial exposure; (2) concentration-dependent bacterial killing during peak cefquinome exposure; (3) time-dependent bacterial regrowth during drug elimination; and (4) an inoculum-dependent reduction in antibacterial efficacy. These findings demonstrate the robustness of the semi-mechanistic PK/PD model for characterizing cefquinome activity and support its application in rational dosing-regimen optimization. Furthermore, the integrated ex vivo–in vivo PK/PD framework demonstrated that the antibacterial activity of cefquinome against *Streptococcus suis* serotype 2 is primarily driven by time-dependent drug exposure, strongly influenced by the initial bacterial inoculum, and reduced in resistant phenotypes. Integrating pharmacokinetic profiles into a mechanistic growth–kill model enabled accurate simulation of bacterial population dynamics across varying exposure conditions and provided a quantitative framework for the rational optimization of cefquinome dosing strategies.

## 4. Discussion

*Streptococcus suis* serotype 2 is an emerging zoonotic pathogen responsible for severe invasive diseases, including septicemia and meningitis, in both humans and pigs [20]. Because bloodstream infections are strongly influenced by circulating drug levels, achieving and maintaining effective serum concentrations is critical for successful treatment in conditions such as sepsis and septic shock [21]. Consequently, serum-based ex vivo models provide a relevant platform for evaluating antimicrobial activity under physiologically meaningful conditions [19,22].

Ex vivo systems using serum or tissue cage fluids have been widely applied to assess the antibacterial activity of antimicrobial agents [23]. However, their interpretation is often limited by the absence of robust mechanistic models capable of describing time-dependent drug effects, particularly for antibiotics whose activity correlates with the PK/PD index %T>MIC [24]. Traditional exposure indices such as AUC24h/MIC have shown inconsistent predictive performance and may not adequately capture treatment outcomes for β-lactams [25]. Even modified approaches such as weighted AUC still rely on specific dosing assumptions and are not universally applicable across regimens [19]. Therefore, a more comprehensive model- and simulation-based PK/PD framework was employed in the present study to characterize cefquinome exposure-response relationships.

The major novelty of this study is the application of a mechanistic PK/PD modeling approach to cefquinome therapy against *S. suis* serotype 2 that goes beyond traditional MIC-based indices. Previous studies have characterized cefquinome activity using PK/PD targets such as %T>MIC and demonstrated the influence of inoculum size on antimicrobial efficacy [26,27]. However, these approaches provide limited information regarding the temporal relationship between drug exposure and bacterial response. In contrast, the present model incorporates bacterial growth dynamics, maximum killing capacity, drug concentration–effect relationships, and delayed antimicrobial responses, allowing prediction of bacterial trajectories under clinically relevant dosing scenarios. A second important advance is the simultaneous evaluation of normal-inoculum, high-inoculum, and reduced susceptibility conditions within a unified modeling framework. Although antimicrobial resistance is commonly considered the major determinant of treatment failure, our findings demonstrate that bacterial burden may have an equal or greater influence on cefquinome efficacy. This observation highlights a limitation of resistance-based MIC interpretation alone, because identical susceptibility categories may produce different therapeutic outcomes depending on infection density and bacterial physiological state. Furthermore, the integration of ex vivo serum time-kill data with in vivo cefquinome pharmacokinetics provides a translational bridge between laboratory observations and clinical dosing optimization. Similar model-based approaches have been successfully applied to other antimicrobial agents to improve dose selection and overcome limitations associated with conventional PK/PD indices [28,29,30].

Unlike MIC-based approaches, which rely on single time-point endpoints (e.g., 24 or 72 h), model-based PK/PD methods describe the full dynamic interaction between drug and bacteria over time by integrating time-kill data with pharmacokinetic profiles [31]. This approach allows simulation of bacterial responses under multiple dosing scenarios. As previously demonstrated, identical endpoint bacterial counts may mask fundamentally different dynamic behaviors, such as rapid killing followed by regrowth, which cannot be distinguished using MIC-based analysis alone [28].

In the present study, a semi-mechanistic model incorporating logistic bacterial growth, a sigmoidal Emax killing function, nutrient limitation, and a time-delay component was successfully applied to describe cefquinome ex vivo activity. This framework yielded key pharmacodynamic parameters (k_0_, k_max_, and EC_50_) that quantified bacterial growth and drug-mediated killing. The estimated growth rates (k_0_) in serum were lower than those typically observed in nutrient-rich in vitro systems, likely reflecting the more limited nutritional environment of serum compared with media such as TSB. The maximal killing rate (kmax) was highest in the NI group (1.39 ± 0.24 h^−1^) and lowest in the HI group (0.78 ± 0.19 h^−1^), demonstrating that increased bacterial burden substantially reduced the rate of cefquinome-mediated killing. Importantly, interpretation of the resistant mutant should consider multiple pharmacodynamic parameters rather than kmax alone. The MS group exhibited a four-fold higher MIC than the HI group (0.24 vs. 0.06 µg/mL) and a higher EC_50_ than the NI group (0.15 vs. 0.08 µg/mL), indicating reduced susceptibility to cefquinome. Although the estimated k_max_ for the MS group (0.99 ± 0.18 h^−1^) was higher than that observed under HI conditions, this parameter reflects the maximal killing capacity once sufficient drug exposure is achieved and does not account for the substantially higher concentration required to achieve that effect. Thus, the combined MIC, EC_50_, and kmax profiles indicate that both bacterial susceptibility and inoculum size influenced cefquinome activity, with high inoculum primarily reducing the achievable killing rate and the resistant phenotype increasing the exposure required for effective bacterial killing.

A major advantage of the present PK/PD framework was its ability to predict bacterial responses under different dosing regimens by integrating pharmacokinetic inputs. In the NI group, all regimens produced measurable initial antibacterial activity; however, sustained suppression differed according to the dosing interval, and 2 mg/kg every 24 h was sufficient to maintain effective bacterial suppression over three days. Although %T>MIC values exceeded the commonly accepted threshold for β-lactams (>40%) in all regimens [29], this MIC-based prediction did not fully align with dynamic simulation results.

In the MS group, 2 mg/kg every 24 h (%T>MIC = 37.5%) and 4 mg/kg every 24 h (%T>MIC = 45.8%) both resulted in bacterial regrowth, despite meeting or approaching conventional PK/PD targets. In contrast, shortening the dosing interval markedly improved outcomes (%T>MIC ≈ 75%), producing sustained bactericidal activity. These discrepancies highlight a key limitation of MIC-based indices, which do not account for post-exposure regrowth dynamics or time-dependent bacterial recovery beyond the standard 24 h endpoint. Indeed, %T>MIC thresholds above 40% may be sufficient for static or limited killing effects but not necessarily for sustained bactericidal activity [32].

In the HI group, bacterial killing was slower and less pronounced, even with increased exposure or shortened dosing intervals. Compared with the MS group, high-inoculum conditions exhibited a blunted response to cefquinome, suggesting that bacterial burden plays a more critical role than resistance level in modulating drug efficacy. Given that cefquinome is a cell wall-active agent, its efficacy depends on active bacterial growth, and reduced growth rates under high-density conditions may impair target engagement [33]. Therefore, high inoculum represents a greater therapeutic challenge than moderate resistance in the context of cefquinome dosing optimization.

Model- and simulation-based approaches have been increasingly recognized as superior to MIC-based methods for optimizing antimicrobial therapy [28]. For example, PK/PD modeling has been successfully used to refine dosing strategies in complex clinical settings, including renal impairment and dialysis populations [34]. Similarly, comparative analyses have demonstrated that integrating multiple modeling approaches improves dosing precision and supports antimicrobial development [30]. These findings reinforce the importance of quantitative, time-resolved PK/PD frameworks in guiding rational antibiotic use in clinical practice.

The stronger influence of inoculum size compared with moderate resistance observed in this study may reflect several biological mechanisms associated with the inoculum effect. High bacterial density can reduce β-lactam efficacy through multiple processes, including increased bacterial biomass requiring greater antimicrobial exposure, altered metabolic activity, reduced bacterial growth rates, and the presence of phenotypically tolerant subpopulations. Because β-lactam antibiotics such as cefquinome exert their activity primarily through inhibition of actively dividing bacterial cells, reduced growth activity under high-density conditions may decrease the rate of cell wall synthesis and subsequently limit antibiotic-mediated killing [28,35]. In addition, high-inoculum populations may generate increased amounts of β-lactamase enzymes or other protective mechanisms that reduce effective drug concentrations at the bacterial target site. The inoculum effect has been widely described for β-lactam antibiotics and has been associated with reduced clinical efficacy despite apparent susceptibility according to standard MIC testing [5,36]. Interestingly, in the present study, the high-inoculum condition produced greater impairment of cefquinome activity than the resistant mutant despite the mutant exhibiting a higher MIC. This observation suggests that MIC alone may not fully capture the biological complexity influencing treatment response. Instead, bacterial burden and physiological state may substantially modify antimicrobial effectiveness, emphasizing the importance of incorporating dynamic bacterial behavior into PK/PD models.

The dosing regimens identified in this study should be regarded as model-based predictions rather than definitive clinical dosing recommendations. The simulations were derived from an ex vivo serum PK/PD model and therefore do not fully account for host immune responses, tissue-specific drug distribution, infection-site physiology, or other factors that may influence treatment outcome in vivo. Accordingly, the predicted regimens require validation in controlled infection models and larger pharmacokinetic studies before their clinical applicability can be established.

Several limitations of the present study should be acknowledged. First, the pharmacokinetic evaluation included a relatively small number of animals (*n* = 8), which may limit the characterization of inter-individual variability in cefquinome disposition. Although the two-compartment model adequately described the observed concentration–time profiles, larger pharmacokinetic studies involving animals with a broader range of physiological characteristics would provide more robust estimates of population variability and improve model generalizability.

Second, antibacterial activity was evaluated using an ex vivo serum model. This approach provides valuable information regarding drug exposure–response relationships under physiologically relevant serum conditions; however, ex vivo systems cannot fully reproduce the complexity of an infected host, including immune responses, tissue penetration, inflammatory processes, and dynamic changes in bacterial populations during infection. In addition, cefquinome protein binding was not directly quantified in the present study; therefore, the PK/PD analyses were based on total serum concentrations rather than directly measured unbound concentrations. Consequently, the predicted dosing strategies should be considered model-based predictions and require further evaluation in controlled experimental infection models before clinical application [17].

Importantly, serum drug concentrations do not necessarily reflect drug exposure at specific sites of infection. This consideration is particularly relevant to cefquinome, as differences between serum and tissue-fluid exposure have been demonstrated following administration in pigs. Cefquinome concentrations and pharmacokinetic profiles in tissue-cage fluid differed from those observed in serum, indicating that systemic serum concentrations may not fully represent local tissue exposure [17,37]. Similarly, physiologically based pharmacokinetic modeling in pigs has demonstrated differences between plasma and lung interstitial-fluid exposure, further highlighting the importance of tissue-specific drug distribution when evaluating antimicrobial efficacy at localized infection sites [38]. Therefore, the present serum-based PK/PD model is most directly relevant to systemic exposure and serum-mediated antibacterial activity and should not be assumed to predict efficacy equally well for localized or tissue-associated infections.

The ex vivo model also does not reproduce biofilm-associated infections, in which altered bacterial physiology, extracellular matrix formation, reduced growth rates, and restricted antimicrobial penetration can decrease antimicrobial susceptibility [39,40]. Accordingly, the present model should not be extrapolated directly to biofilm-associated infections without additional experimental validation. These considerations reinforce the need for subsequent validation in appropriate in vivo infection models and, where relevant, tissue-specific PK/PD studies.

Third, only one cefquinome-resistant mutant was evaluated. Although M1 provided a useful model of reduced cefquinome susceptibility, naturally occurring resistant isolates may possess diverse resistance mechanisms and different fitness characteristics. Moreover, whole-genome sequencing or targeted genetic analyses were not performed; therefore, the specific genetic basis of the increased cefquinome MIC in M1 could not be established. Future studies should include multiple resistant clinical isolates with characterized resistance mechanisms to determine whether the observed PK/PD relationships are applicable across different resistance phenotypes.

Fourth, the developed model was internally evaluated using the experimental datasets generated in the present study; however, external validation using independent bacterial strains, additional infection models, or clinical outcome data was not performed. Therefore, although the model demonstrated good descriptive performance within the current experimental framework, its predictive performance and generalizability require confirmation in independent datasets and in vivo infection models before broader application for cefquinome dose optimization.

Finally, both male and female piglets were included in the study in equal numbers (four males and four females). However, the sample size was not sufficient to provide adequate statistical power for a formal assessment of sex-related differences in cefquinome pharmacokinetics or pharmacodynamics. Because the crossover design resulted in all eight animals receiving both cefquinome doses, the dose groups should not be considered independent sex-stratified groups. Therefore, PK and ex vivo PD results were analyzed in aggregate rather than stratified by sex. Although sex-related differences cannot be excluded, substantially larger studies would be required to determine whether sex significantly influences cefquinome disposition or pharmacodynamic response. Future population PK/PD studies should consider sex as a prespecified covariate when sufficient sample size is available.

In summary, this study demonstrates that a model- and simulation-based PK/PD approach provides a robust framework for describing cefquinome activity against *S. suis* serotype 2 across different inocula, MIC levels, and dosing regimens. The model accurately captured time-kill dynamics and enabled prediction of optimal dosing strategies beyond what is achievable using MIC-based indices alone. Specifically, 2 mg/kg every 24 h for 3 days was sufficient for infections with normal inocula, whereas 2 mg/kg every 12 h was required for high-inoculum infections and moderately resistant strains (MIC ≤ 0.24 µg/mL). Notably, slow killing was predominantly associated with high-inoculum conditions, underscoring its major impact on therapeutic outcome. Overall, these findings highlight the utility of model-based PK/PD approaches as a powerful and flexible tool for optimizing antibiotic dosing regimens and complementing conventional MIC-based evaluation. Although the simulated dosing regimens identified in this study provide valuable insight into cefquinome exposure requirements, these results should be interpreted cautiously. The proposed regimens are based on mechanistic PK/PD simulations derived from ex vivo serum experiments and should not be considered direct clinical dosing recommendations. Translation into veterinary practice requires confirmation through controlled infection models, larger pharmacokinetic studies, and ultimately clinical efficacy investigations. Nevertheless, the current findings provide a quantitative framework for guiding future cefquinome optimization studies and demonstrate the potential value of model-informed approaches for improving antimicrobial therapy.

## 5. Conclusions

This study demonstrates that cefquinome exhibits time-dependent antibacterial activity against *Streptococcus suis* serotype 2, with efficacy strongly influenced by bacterial inoculum size and susceptibility. Ex vivo serum time-kill experiments revealed a pronounced inoculum effect, with higher bacterial burdens associated with reduced maximal killing and enhanced regrowth. The cefquinome-resistant mutant also exhibited reduced susceptibility and attenuated antibacterial response compared with the parental strain.

A semi-mechanistic PK/PD model incorporating bacterial growth, sigmoidal Emax-mediated killing, nutrient limitation, and delayed drug effects adequately described the observed ex vivo time-kill dynamics and integrated in vivo cefquinome pharmacokinetic profiles. The model captured important features of the drug–bacteria interaction, including delayed killing, regrowth during declining drug exposure, and reduced antibacterial activity under high-inoculum conditions. These dynamic responses were not fully characterized by conventional MIC-based %T>MIC analysis based on total serum cefquinome concentrations.

Model-based simulations indicated that 2 mg/kg every 24 h provided effective bacterial suppression under normal-inoculum conditions, whereas more frequent administration at 2 mg/kg every 12 h improved antibacterial activity under high-inoculum and reduced-susceptibility conditions. Notably, bacterial burden appeared to exert a greater influence on treatment response than the moderate reduction in susceptibility represented by the M1 mutant. These findings support the value of integrating PK data with time-resolved bacterial response models to better characterize cefquinome exposure–response relationships.

Overall, the present model- and simulation-based PK/PD framework provides a quantitative and mechanistically informative approach for evaluating cefquinome dosing strategies against *S. suis* serotype 2. The predicted regimens, including 2 mg/kg every 24 h for normal-inoculum conditions and 2 mg/kg every 12 h for high-inoculum or reduced-susceptibility conditions, should be regarded as model-based, hypothesis-generating predictions rather than direct clinical dosing recommendations. Further validation in controlled in vivo infection models, larger pharmacokinetic studies, and ultimately clinical efficacy studies is required before these dosing strategies can be translated into veterinary practice.

## Figures and Tables

**Table 1:** Pharmacokinetic parameters of cefquinome in serum following intramuscular administration at doses of 2 and 4 mg/kg in piglets. Pharmacokinetic parameters were estimated using a two-compartment model. A and B represent the *y*-axis intercepts for the distribution and elimination phases, respectively. t_1/2α_ indicates the distribution half-life, while t_1/2β_ represents the elimination half-life. K_10_ denotes the elimination rate constant from the central compartment. K_12_ and K_21_ represent the transfer rate constants between the central and peripheral compartments. AUC refers to the area under the serum concentration–time curve. Vss represents the volume of distribution at steady state, and CL indicates total body clearance. C_max_ is the maximum serum concentration, whereas T_max_ represents the time required to reach C_max_.

Parameters (Units)	Regimen (Mean ± SD)	
	2 mg/kg (*n* = 8)	4 mg/kg (*n* = 8)
A (μg/mL)	4.48 ± 3.23	5.62 ± 2.16
B (μg/mL)	4.53 ± 0.56	7.25 ± 1.14
t_1/2α_ (h)	0.24 ± 0.05	0.71 ± 0.14
t_1/2β_ (h)	2.13 ± 0.10	2.22 ± 0.21
K_10_ (h^−1^)	0.51 ± 0.10	0.44 ± 0.03
K_12_ (h^−1^)	0.89 ± 0.58	0.49 ± 0.32
K_21_ (h^−1^)	1.87 ± 0.21	1.03 ± 0.93
AUC (μg·h/mL)	14.53 ± 1.63	28.11 ± 2.62
Vss (L/kg)	0.12 ± 0.06	0.07 ± 0.04
CL (L/h/kg)	0.14 ± 0.01	0.14 ± 0.02
C_max_ (μg/mL)	5.70 ± 0.76	10.86 ± 0.97
T_max_ (h)	0.20 ± 0.02	0.17 ± 0.04

**Table 2:** Pharmacodynamic parameters and goodness-of-fit criteria for *Streptococcus suis* serotype 2. k_0_ represents the bacterial growth rate constant in the absence of cefquinome, while k_max_ denotes the maximum bacterial killing rate constant (maximum effect). EC_50_ is the cefquinome concentration required to achieve 50% of the maximum antibacterial effect. N_max_ indicates the maximum bacterial population size, h represents the Hill coefficient, and γ corresponds to the constant used to describe the initial lag phase associated with bacterial inhibition or killing. MSC refers to the model selection criterion used to evaluate goodness of fit. ND, not determined; NI, normal inoculum; HI, high inoculum; MS, mutant strain.

Parameters	NI (Mean ± SD)	HI (Mean ± SD)	MS (Mean ± SD)
k_0_ (h^−1^)	0.67 ± 0.09	0.32 ± 0.03	0.55 ± 0.03
k_max_ (h^−1^)	1.39 ± 0.24	0.78 ± 0.19	0.99 ± 0.18
EC_50_ (µg/mL)	0.08 ± 0.06	0.18 ± 0.02	0.15 ± 0.03
N_max_	1.66 × 10^7^	1.8 × 10^8^	2.15 × 10^7^
h	1.2 ± 0.2	0.6 ± 0.25	1.54 ± 0.32
γ	ND	1.2 ± 0.13	2.308 ± 0.15
MSC/R^2^	2.9/0.996	1.617/0.986	1.57/0.993

**Table 3:** Mean %T>MIC values for the three cefquinome dosing regimens evaluated against *Streptococcus suis* serotype 2. %T>MIC represents the percentage of the dosing interval during which cefquinome concentrations remained above the minimum inhibitory concentration (MIC). NI, normal inoculum; HI, high inoculum; MS, mutant strain.

	%T>MIC (Mean)		
Different Infection Events	2 mg/kg q 12 h	2 mg/kg q 24 h	4 mg/kg q 24 h
NI (MIC: 0.03 µg/mL)	100.00	62.50	70.83
HI (MIC: 0.06 µg/mL)	100.00	54.16	64.58
MS (MIC: 0.24 µg/mL)	75.00	37.50	45.83

## Data Availability

The original contributions presented in this study are included in the article. Further inquiries can be directed to the corresponding author.

## Abbreviations

Abbreviation	Full term
AUC	Area under the concentration–time curve
AUC24h	Area under the concentration–time curve over 24 h
AIC	Akaike information criterion
β-lactams	Beta-lactam antibiotics
CFU	Colony-forming units
CL	Clearance
CLSI	Clinical and Laboratory Standards Institute
C_max_	Maximum plasma concentration
EC_50_	Concentration producing 50% of maximal effect
E_max_	Maximum effect model
HI	High-inoculum group
IM	Intramuscular
MIC	Minimum inhibitory concentration
MSC	Model selection criterion
MS	Mutant strain (cefquinome-resistant derivative)
NI	Normal inoculum group
PK	Pharmacokinetics
PD	Pharmacodynamics
PK/PD	Pharmacokinetic/pharmacodynamic
q12h	Every 12 h
q24h	Every 24 h
R^2^	Coefficient of determination
*S. suis*	Streptococcus suis
T>MIC	Time during which drug concentration exceeds MIC
TSB	Tryptic soy broth
k_0_	Bacterial growth rate constant
k_max_	Maximum bacterial killing rate
γ	Lag time parameter for drug effect onset
